# Fathers’ Role in Bulimia Nervosa: A Systematic Review

**DOI:** 10.1192/j.eurpsy.2023.913

**Published:** 2023-07-19

**Authors:** M. N. Akkese, J. Treasure, H. Himmerich

**Affiliations:** 1 PO59 Section of Eating Disorders, Department of Psychological Medicine, Institute of Psychiatry, Psychology & Neuroscience, King’s College London, London, SE5 8AF; 2 Eating Disorders Unit, Bethlem Royal Hospital, South London and Maudsley NHS Foundation Trust (SLaM), Maudsley Hospital, Denmark Hill, London, SE 8AZ, United Kingdom

## Abstract

**Introduction:**

Bulimia Nervosa (BN) is a highly prevalent eating disorder related to multiple risk factors. In this regard, familial variables can play a critical role in the development and maintenance of BN.

**Objectives:**

The existing studies frequently explored mothers and maternal factors, while fathers and paternal variables have been less extensively investigated in this field. Therefore, we aimed to systematically review the studies on the role of paternal factors in BN.

**Methods:**

This systematic review process was carried out according to the Preferred Reporting Items for Systematic Reviews and Meta-Analysis (PRISMA) guidelines. As a result of the literature search on PubMed, Web of Science TM, and APA PsycINFO, 419 candidate papers were determined and evaluated based on the eligibility criteria. The quality assessment of the final 59 studies was conducted using the JBI Critical Appraisal Tools.

**Results:**

Then, we thematically arranged and narratively reported the qualitative and quantitative research findings. Paternal attitudes (e.g., critical, abusive, aggressive, uncaring, and unaffectionate), family dynamics (e.g., chaotic, rigid, less communicative, and emotionally involved), and father-specific features (e.g., personality traits, eating psychopathology features) were found as three main groups that could be directly or indirectly associated with the development and maintenance of BN symptoms. The eligible qualitative studies also indicated that fathers could positively influence the recovery process of their daughters with BN (e.g., by helping them develop healthy adaptive body image, self-adequacy, and self-esteem).

**Conclusions:**

The contradictory outcomes were discussed for further research and clinical implications.

**Disclosure of Interest:**

None Declared

